# Sight Unseen: Glucagon-Like Peptide-1 (GLP-1) Agonism Therapy and Nonarteritic Anterior Ischemic Optic Neuropathy

**DOI:** 10.7759/cureus.100953

**Published:** 2026-01-06

**Authors:** Maxim J Barnett, Festus Ibe, Justin Lam

**Affiliations:** 1 Internal Medicine, Jefferson Einstein Philadelphia Hospital, Philadelphia, USA

**Keywords:** anterior ischaemic optic neuropathy, glp-1 ra, glucagon-like peptide-1 receptor agonist, ischemic optic neuropathy, non-artertic anterior ischemic optic neuropathy (naion), optic neuropathy

## Abstract

Introduction

Nonarteritic anterior ischemic optic neuropathy (NAION) is one of the most common causes of blindness among adults. Numerous risk factors have been noted (such as systemic diseases and medications); however, the underlying pathophysiology has yet to be elucidated. In recent years, however, glucagon-like peptide-1 (GLP-1) receptor agonists have emerged as a cause of NAION, albeit evidence is mixed. As a result, the primary objective of this study is to further analyze the risk of NAION over a five-year period in type 2 diabetes mellitus patients treated with GLP-1 receptor agonists.

Methods

We utilized TriNetX Global Collaborative Network to perform a retrospective cohort analysis, using de-identified patient data. Patients were divided into two cohorts (cohort A: type 2 diabetes mellitus with GLP-1 treatment; cohort B: type 2 diabetes mellitus without exposure to GLP-1). One-to-one propensity-score matching was employed for n = 20 covariates. We employed a measure of association, calculating risk difference, risk ratio, and 95% confidence interval. Furthermore, those with the outcome of interest (NAION) prior to the index event were excluded from analysis.

Results

After propensity-score matching, we arrived at n = 388,333 per cohort (n = 776,666 total). Over a five-year period, cohort A demonstrated a statistically significant increased risk of NAION (risk difference 0.022%, 95% CI 0.01%-0.034%; risk ratio 1.339, 95% CI 1.137-1.577, p = 0.005). Additionally, E-value sensitivity analysis confirmed a moderately robust association.

Conclusion

Whilst of statistical significance, the implicated clinical significance is less concrete, due to a well-established strong benefit-to-risk ratio with the therapeutic class. The exact mechanism of NAION in relation to GLP-1 therapy remains unknown. Further prospective studies are required to evaluate such findings and adjust existing guidelines as appropriate.

## Introduction

Glucagon-like peptide-1 (GLP-1) is an incretin hormone released by L-cells in the intestine in response to nutrient intake, functioning to enhance glucose-dependent insulin secretion, suppress glucagon release, slow gastric emptying, and reduce appetite [1]. The hormone itself was initially identified in 1986 in Denmark, with further identification of exendin from the Gila monster saliva. Subsequently, the first GLP-1, exenatide, reached the market in 2005 (after approval from the Food and Drug Administration) [2]. Unlike physiological GLP-1, which has a half-life of two to three minutes, exogenous administration bypasses inactivation by the enzyme dipeptidyl-peptidase IV, allowing GLP-1 to reach levels 100 times normal [3].

The GLP-1 receptor agonists have made many advances since their debut 20 years ago, including the reduction from twice daily to daily injection. An oral form of semaglutide has also been developed. Additionally, dual incretin (GLP-1 and gastric-inhibitory-polypeptide (GIP)) agonism with tirzepatide has allowed for even greater efficacy. Current ongoing trials are now investigating triple agonism with examples including retatrutide, targeting GLP-1, GIP, and glucagon receptors [4]. While GLP-1s were initially approved for glycemic control in type 2 diabetes mellitus, their benefits extend beyond glycemic control, demonstrating multisystemic effects; examples include neuroprotection, nephroprotection, cardiovascular benefits, weight loss, obstructive sleep apnea, and anti-fibrosis within the liver, among others [5]. Due to the widespread benefits, most notably weight loss, there has been a significant increase in prescriptions. As an example, in 2023, semaglutide was the most commonly prescribed medication within the United States, with just below 2% of the population receiving this medication [6,7]. Additionally, between the years 2021 and 2023, there was an estimated 60% increase in the United States in new-to-brand prescriptions for semaglutide [8].

The GLP-1 class of medications is not without adverse events, notably gastrointestinal symptoms, ranging from self-limiting nausea and vomiting to pancreatitis, warranting discontinuation. Notably, in those with a personal or family history of medullary thyroid carcinoma, GLP-1 medications are contraindicated, due to the theoretical risk for medullary thyroid cancer (which has not been proven in human subjects) [9]. Within the last two years, however, adverse ocular events have been increasingly recognized, namely, nonarteritic anterior ischemic optic neuropathy (NAION) [9].

While there have been conflicting reports within the literature confirming or refuting this latter association, we perform a retrospective cohort analysis and provide a literature review to further review this phenomenon.

## Materials and methods

Study design

We performed a retrospective cohort analysis using de-identified patient data through TriNetX Global Collaborative Network, providing access to 200 million anonymized electronic health records and over 140 healthcare organizations. With the utilization of de-identified data, our study was exempt from an institutional review board. Our study was designed to address the primary objective of comparing the risk of NAION among patients with type 2 diabetes mellitus who are prescribed GLP-1 therapy, over those who are not.

Inclusion criteria for this study included both male and female genders of at least 18 years of age, in both inpatient and outpatient settings. Furthermore, there was no geographic restriction; although TriNetX is globally available in 21 countries, the majority of patients (in excess of 90%) are within the United States. For adequate identification of diagnoses, we utilized the 10th revision of the International Statistical Classification of Diseases and Health Problems (ICD-10) codes; for identification of medication classes, we utilized the United States National Library of Medicine (RxNorm) codes within the TriNetX platform. Patients must have held a diagnosis of type 2 diabetes mellitus (ICD-10: E11) as well as an HbA1c of at least 6.5%. Cohorts were stratified based on the administration of GLP-1 therapy (RxNorm A10BJ) (Cohort A) or lack of exposure to GLP-1 therapy (RxNorm A10BJ) (Cohort B). The outcome in question was NAION (ICD-10: H47.01).

Statistical analysis

We defined the index event as the first date a patient was eligible for inclusion into either Cohorts A or B. In addition, a time interval of five years was incorporated to assess the development of the outcome of interest (NAION). To prevent untoward selection bias, patients with the outcome of interest (NAION) prior to the defined index event were excluded from analysis among both cohorts.

A one-to-one propensity-score matching was incorporated to minimize confounding (using the greedy/nearest neighbor matching algorithm with 0.1 caliper of pooled standard deviations) allowing for balance among Cohorts A and B. Patients were matched for 20 variables, including age; gender; ethnicity; hyperlipidemia (ICD-10 E78.5); hypertension (ICD-10: I10-I1A); chronic kidney disease (ICD-10: N18); oral hypoglycemic agents (RxNorm: HS502); insulin (RxNorm: HS501); HbA1c; body mass index (BMI); other disorders of the eye and adnexa (ICD-10: H57); type 2 diabetes mellitus with unspecified diabetic retinopathy without macular edema (ICD-10: E11.319); type 2 diabetes mellitus with unspecified diabetic retinopathy with macular edema (ICD-10: E11.311); type 2 diabetes mellitus with ophthalmic complications (ICD-10: E11.3). Covariates were prespecified and selected based on prior literature and clinical relevance (rather than unadjusted statistical associations). 

Measures of association were estimated using absolute risk differences and relative risks (rather than odds ratios), which are appropriate for binary outcomes (and provide clinically interpretable effect estimates). Analyses were performed before and after propensity-score matching. Statistical inference was based on two-sided hypothesis testing with alpha set at 0.05. While full model-based outputs were generated at the time of analysis, certain results are reported with p-values only because the original analytical output files were not retained (precluding extraction of confidence intervals for those specific estimates, although this does not affect direction or statistical significance of the findings).

We subsequently proceeded to perform a sensitivity analysis using an E-value measurement to assess for potential unmeasured confounds that may influence the outcome of interest; an E-value above 2.0 was considered moderately robust, and above 3.0 strongly robust. Statistical analyses were performed through the TriNetX platform.

## Results

Our initial search identified n = 107 healthcare organizations. Prior to matching, Cohort A demonstrated n = 643,364, with Cohort B demonstrating n = 1,784,568. Following propensity-score matching, we identified a total of n = 776,666 (n = 388,333 per cohort). After matching, Cohort A demonstrated a mean age of 63 + 12.8 compared to 62.5 + 15.9 (p < 0.0001), white (60.104% versus 60.813%, p < 0.0001), mean HbA1c (8.34% + 1.99 versus 7.99% + 1.96, p < 0.0001), and mean BMI (35 + 8.04 versus 33.6 + 8.2, p < 0.0001) (Table 1).

**Table 1:** Cohort Characteristics Before and After Propensity-Score Matching Abbreviations: HbA1c = Glycated Hemoglobin; SD = Standard Deviation; Std. Diff = Standard Difference

Variables	Before Matching							After Matching							
	Cohort A	Cohort B	Patients (n) (Cohort A)	Patients (n) (Cohort B)	% of Cohort (Cohort A)	% of Cohort (Cohort B)	p-value	Cohort A	Cohort B	Patients (n) (Cohort A)	Patients (n) (Cohort B)	% of Cohort (Cohort A)	% of Cohort (Cohort B)	p-value	Std. Diff.
Current Age ± SD	61.3 ± 13.1	66.8 ± 14.9	643,364	1,784,568	100%	100%	< 0.0001	63 ± 12.8	62.5 ± 15.9	388,333	388,333	100%	100%	< 0.0001	0.0385
Age at Index ± SD	58.1 ±- 13.1	62.7 ± 14.9	643,364	1,784,568	100%	100%	< 0.0001	59.4 ± 12.8	58.7 ± 15.8	388,333	388,333	100%	100%	< 0.0001	0.0502
White	-	-	399,685	949,773	62.124%	53.221%	< 0.0001	-	-	233,403	236,156	60.104%	60.813%	< 0.0001	0.0145
Black or African American	-	-	115,476	288,605	17.949%	16.172%	< 0.0001	-	-	68,.524	70,458	17.646%	18.144%	< 0.0001	0.0130
Not Hispanic or Latino	-	-	421,861	1,092,168	65.571%	61.201%	< 0.0001	-	-	250,248	247,977	64.442%	63.857%	< 0.0001	0.0122
Unknown Ethnicity	-	-	162,696	533,349	25.288%	29.887%	< 0.0001	-	-	102,262	103,190	26.334%	26.573%	0.0170	0.0054
Unknown Race	-	-	61,983	305,452	9.634%	17.116%	< 0.0001	-	-	43,232	39,555	11.133%	10.186%	< 0.0001	0.0307
Female	-	-	331,267	781,383	51.49%	43.786%	< 0.0001	-	-	192,665	194,239	49.613%	50.019%	0.0004	0.0081
Male	-	-	281,189	937,576	44.794%	52.538%	< 0.0001	-	-	181,263	179,455	46.677%	46.212%	< 0.0001	0.0093
Hypertension	-	-	506,086	1,103,388	78.662%	61.829%	< 0.0001	-	-	287,514	277,429	74.038%	71.441%	< 0.0001	0.0583
Hyperlipidemia	-	-	397,915	733,544	61.849%	41.105%	< 0.0001	-	-	216,196	208,930	55.673%	53.802%	< 0.0001	0.0376
Chronic Kidney Disease	-	-	120,206	285,282	18.684%	15.986%	< 0.0001	-	-	70,673	68,736	18.199%	17.7%	< 0.0001	0.0130
Type 2 Diabetes Mellitus With Unspecified Diabetic Retinopathy Without Macular Edema	-	-	37,378	56,164	5.81%	3.147%	< 0.0001	-	-	20,480	21,509	5.274%	5.539%	< 0.0001	0.0117
Type 2 Diabetes Mellitus With Unspecified Diabetic Retinopathy With Macular Edema	-	-	21,760	34,290	3.382%	1.921%	< 0.0001	-	-	12,896	13,549	3.321%	3.489%	< 0.0001	0.0026
Type 2 Diabetes Mellitus With Ophthalmic Complications	-	-	78,662	108,528	12.227%	6.081%	< 0.0001	-	-	38,919	39,859	10.022%	10.264%	0.0004	0.0093
Other Disorders of the Eye and Adnexa	-	-	31,286	41,021	4.863%	2.299%	< 0.0001	-	-	14,601	14,792	3.76%	3.809%	0.0026	0.0026
Oral Hypoglycemic Agents	-	-	518,229	710,742	80.55%	39.827%	< 0.0001	-	-	272,837	264,196	70.259%	68.033%	< 0.0001	0.0482
Insulin Therapy	-	-	345,685	668.938	53.731%	37.485%	< 0.0001	-	-	194,797	194,697	50.162%	50.137%	0.8205	0.0005
HbA1c	8.39 ±1.94	7.63 ± 1.95	614,047	968,233	95.443%	54.256%	< 0.0001	8.34 ± 1.99	7.9 ± 1.96	359.208	327,775	92.5%	84.406%	< 0.0001	0.2254
Body Mass Index	36 ± 8.16	31.6 ± 7.59	497,915	1,083,208	77.392%	60.699%	< 0.0001	35 ± 8.04	33.6 ± 8.2	284,332	289,665	73.219%	74.592%	< 0.0001	0.1703

**Figure 1:**
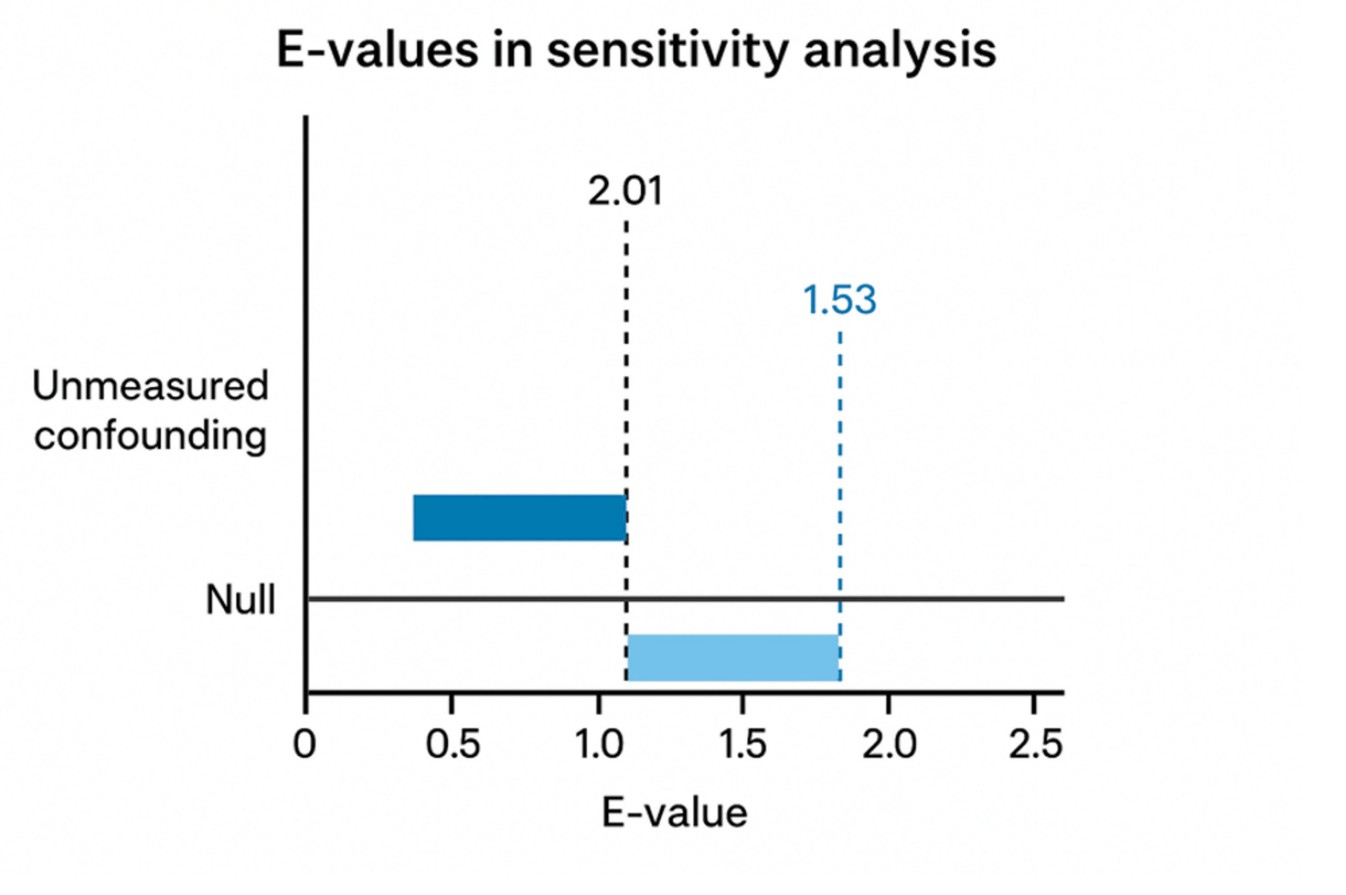
E-Value Sensitivity Analysis Sensitivity analysis was performed with an E-value. An E-value point estimate was measured at 2.01, meaning the observed association is moderately robust; an unmeasured confounder would need to be associated with exposure and outcome by a relative risk of at least 2.01 to fully explain away the observed association. An E-value interval limit of 1.53 was calculated, suggesting an unmeasured confounder needs to be associated with both exposure and outcome by a relative risk of 1.53 to move the confidence interval towards the null, whereby there is no overall effect.

Our data demonstrated a higher incidence of NAION in Cohort A (0.087%) compared to Cohort B (0.065%) over a five-year period, with a risk difference of 0.022% (95% CI 0.01%-0.034%); furthermore, we obtained a risk ratio of 1.339 (95% CI 1.137-1.577) which achieved statistical significance (p = 0.0005). We proceeded with a sensitivity analysis to calculate an E-value with respect to the relative risk, with a point-estimate E-value of 2.01, suggesting that the observed observation is moderately robust, and that an unmeasured confounder would need to be associated with both GLP-1 and NAION by a risk ratio of at least 2.01 to fully explain away the observed observation. Furthermore, the lower CI E-value was measured at 1.53, suggesting an unmeasured confounder would need to be associated with both GLP-1 and NAION by a risk ratio of 1.53 to move the confidence interval to include the null (with no overall effect) (Figure 1).

## Discussion

The first signal of a potential association between semaglutide and NAION emerged from clinical observations at Massachusetts Eye and Ear, whereby neuro-ophthalmologists noted an unusual cluster of NAION among semaglutide users [8]. Since then, numerous studies (including case reports/series, cohort studies, meta-analyses, and review of the FDA Adverse Event Reporting System) have either reported or refuted an association between GLP-1 therapy and NAION (Table 2) [8,10-34].

**Table 2:** Literature Review of Studies Assessing NAION and GLP-1 Abbreviations: CI = Confidence Interval; GLP-1 RA = Glucagon-Like Peptide-1 Receptor Agonist; HR = Hazard Ratio; IRR = Incidence Rate Ratio; NAION = Nonarteritic Anterior Ischemic Optic Neuropathy; OR = Odds Ratio; RR = Relative Risk; USA = United States of America

Author	Country	Design	GLP-1	Result(s)	Interpretation
Katz et al. (2025) [7]	USA	Case Series	Semaglutide and tirzepatide	N/A	Seven cases of NAION.
Hathaway et al. (2024) [8]	USA	Retrospective Cohort Analysis (Single Center)	Semaglutide	T2DM: HR 4.28 (95% CI 1.62-11.29); overweight/obesity: HR 7.64 (95% CI 2.21-26.36)	Statistically significant increased risk of NAION with semaglutide therapy.
Grauslund et al. (2024) [10]	Denmark	Prospective Cohort Analysis (Multicenter)	Semaglutide	HR 2.19 (95% CI 1.54-3.12)	Statistically significant risk predictor for NAION with semaglutide therapy.
Karam et al. (2024) [11]	USA	Case Report	Semaglutide	N/A	Bilateral NAION after less than one year of semaglutide therapy.
Carreno-Galeano et al. (2024) [12]	USA	Retrospective Cohort Analysis (Single Center)	GLP-1 RA	Overall effect: RR 3.24, p < 0.001; Cox regression analysis for T2DM: RR 1.60, p = 0.035	Statistically significant higher risk for NAION with GLP-1 RA.
Simonsen et al. (2025) [13]	Denmark and Norway	Retrospective Cohort Analysis	Semaglutide	HR 2.81; 95% CI 1.67-4.75	Statistically significant increased NAION risk with semaglutide therapy
Chou et al. (2025) [14]	International	Retrospective Cohort Analysis (Multicenter)	Semaglutide	T2DM at 1 year (HR 2.32; 95% CI 0.60-8.97), 2 years (HR 2.31; 95% CI 0.86-6.17), 3 years (HR 1.51; 95% CI 0.71-3.25); T2DM & Obesity at 1 year (HR: 0.81; 95% CI 0.42-1.57), 2 years (HR: 1.2; 95% CI 0.74-1.94) and 3 years (HR: 1.19; 95% CI 0.78-1.82)	No statistically significant increased NAION risk with semaglutide.
Cai et al. (2025) [15]	International	Retrospective Cohort Analysis (Multicenter)	Semaglutide, exenatide, dulaglutide	Semaglutide: IRR 1.32 (95% CI 1.14-1.54) (sensitive definition) and IRR 1.50 (95% CI 1.26-1.79) (specific definition); exenatide: IRR 1.62 (95% CI 1.02-2.58) (specific definition) and IRR 1.26 (95% CI 0.78-2.03) (sensitive definition); dulaglutide: IRR 1.10 (95% CI 0.88-1.39 (sensitive definition); IRR 1.16 (95% CI 0.92-1.45) (specified definition)	Statistically significant increased NAION risk with semaglutide. No statistically significant risk for NAION with dulaglutide. Exenatide demonstrated a statistically significant NAION risk with specific (but not sensitive) definition.
Hsu et al. (2025) [16]	International	Retrospective Cohort Analysis (Multicenter)	Semaglutide	1 year: HR 1.94 (95% CI 0.93-4.02) 2 year: HR 2.39 (95% CI 1.37-4.18) 3 year: HR 2.44 (95% CI 1.44-4.12) 4 year: HR 2.05 (95% CI 1.26-3.34)	Statistically significant increased NAION risk with semaglutide after one year.
Lakhani et al. (2025) [17]	International	Retrospective Pharmacovigilance Analysis (FAERS, VigiBase)	Semaglutide, and tirzepatide	Semaglutide: FAERS ROR 11.12 (95% CI 8.15-15.16); VigiBase ROR 68.58 (95% CI 16.75-280.67); Tirzepatide: FAERS ROR 0.51 (95% CI 0.16-1.58); VigiBase ROR 3.73 (95% CI 0.62-22.32)	Statistically significant disproportionality signal with semaglutide but not tirzepatide.
Procacci et al. (2025) [18]	International	Retrospective Pharmacovigilance Analysis (FAERS)	Semaglutide	Semaglutide: ROR 17.57 (95% CI 13.92-21.90); liraglutide: ROR 1.02 (95% CI 0.38-2.73); GLP-1 RA: ROR 1.98 (95% CI 1.48-2.64)	Statistically significant disproportionality signal with semaglutide.
Azab and Pasina (2025) [19]	International	Retrospective Pharmacovigilance Analysis (FAERS)	GLP-1 RA	ROR 11.36; 95% CI 8.33-15.49	Statistically significant disproportionality signal with semaglutide.
Abbass et al. (2025) [20]	USA	Retrospective Cohort Analysis (Multicenter)	Semaglutide, and GLP-1 RA	Semaglutide: RR 0.7 (95% CI 0.52-0.94); all GLP-1 RR 0.89 (95% CI 0.609-1.102)	No statistically significant increased NAION risk with semaglutide, or any GLP-1 RA.
Andreão et al. (2025) [21]	International	Retrospective Cohort Analysis (Multicenter)	GLP1-RA	RR 0.956 (95% CI 0.629-1.452)	No statistically significant increased NAION risk with GLP-1 RA.
Özbek et al. (2025) [22]	International	Meta-Analysis	GLP1-RA	T2DM: OR 1.41 (95% CI 0.93 -2.16); T2DM and obesity: OR 0.87 (95% CI 0.57-1.33)	No statistically significant increased NAION risk with GLP-1 RA.
Silverii et al. (2025) [23]	International	Meta-Analysis	GLP-1 RA	HR 1.53 (95% CI 0.53-4.44)	No statistically significant increased NAION risk with GLP-1 RA.
Klonoff et al. (2024) [24]	USA	Retrospective Cohort Analysis (Multicenter)	Semaglutide and GLP-1 RA	HR 1.45; 95% CI 0.51-4.17)	No statistically significant increased NAION with semaglutide or GLP-1 RA.
Wang et al. (2025) [25]	USA	Retrospective Cohort Analysis (Multicenter)	Semaglutide, tirzepatide and GLP-1 RA	Semaglutide or tirzepatide compared to other antidiabetic agents: HR 1.76 (95% CI 1.01-3.07); semaglutide or tirzepatide compared to other GLP-1 RA: HR 1.75 (95% CI 0.98-3.10)	Semaglutide/tirzepatide demonstrate a statistically significant increased NAION risk compared to non-GLP-1 RAs; however, there is no statistically significant difference compared to other GLP-1 RA.
Ramsey et al. (2025) [26]	International	Retrospective Cohort Analysis (Multicenter)	GLP-1 RA	HR 1.26 (95% CI 0.94-1.70)	No statistically significant increased NAION risk with GLP-1 RA.
Nagdeve and Griffin (2025) [27]	USA	Case-Control Study (Multicenter)	All GLP-1 receptor agonists, liraglutide, semaglutide	T2DM: GLP-1 RA OR 0.94 (95% 0.85-1.04); dulaglutide OR 0.93 (95% CI 0.75-1.15); exenatide OR 0.83 (95% CI 0.72-0.96); liraglutide OR 1.01 (95% CI 0.86-1.19); semaglutide OR 1.24 (95% CI 0.93-1.66)	No statistically significant increased risk of NAION with GLP-1 RA, dulaglutide, liraglutide or semaglutide. Statistically significant decreased risk of NAION with exenatide.
Natividade et al. (2025) [28]	International	Meta-Analysis	Semaglutide	Semaglutide OR 3.92 (95% CI 1.02-15.02)	Minimal statistically significant increased NAION risk with semaglutide.
Mao et al. (2025) [29]	USA	Case Report	Semaglutide	N/A	N/A
Ahmadi and Hamann (2025) [30]	Denmark	Case Series	Semaglutide	N/A	Four male patients with NAION.
Lixi et al. (2025) [32]	Italy	Case Report	Liraglutide switched to semaglutide	N/A	N/A
Jivraj et al. (2025) [32]	USA	Case Report	Semaglutide	N/A	N/A
Maceroni et al. (2025) [33]	Italy	Case Report	Semaglutide	N/A	N/A
Gaćina et al. (2025) [34]	Croatia	Case Report	Semaglutide	N/A	N/A
Barnett et al. (2025) (Our Study)	International	Retrospective Cohort Analysis	GLP-1RA	RR 1.339 (95% CI 1.137-1.577)	Statistically significant increased NAION risk with GLP-1RA.

Ischemic optic neuropathy (ION) is classified as either anterior or posterior, depending upon the portion of the optic nerve affected [35]. Of these subdivisions, anterior ION accounts for nearly 90% of cases. A further classification of ION involves arteritic (related to vasculitides) and nonarteritic (unrelated to a vasculitic process). Arteritic comprises solely 5-15% of cases and is commonly caused by giant cell arteritis; contrarily, nonarteritic is responsible for up to 95% of cases of ION [36].

NAION is the second most common cause of optic nerve blindness in adults over age 50, after glaucoma [8]. The definition of NAION entails sudden, painless visual loss secondary to a non-vasculitic process leading to ischemia of the optic nerve head [20]. While the diagnosis has gained more attention in recent years, it is certainly not novel, having first been described by Jean-Pierre St. Yves in 1817 [37]. The disease itself is quite rare, estimated to occur between 2.5 and 11.8 per 100,000 cases annually in the United States [38]. NAION appears to be more prevalent among Caucasian males, who are over 50 years of age [39].

The exact underlying pathophysiology is incompletely understood; however, it is believed to result in acute hypoperfusion of the posterior ciliary artery branches of the optic nerve, with subsequent microvascular injury and infarction [36]. Documented risk factors include type 2 diabetes mellitus; vascular spasm; arteriosclerosis; hypertension; smoking; hyperlipidemia; obesity; obstructive sleep apnea; hypercoagulability; nocturnal hypotension; medications (including phosphodiesterase type-5 inhibitors, amiodarone, statins, beta-blockers, anti-thrombotics, cabergoline) [37]. The most consistent risk factor, however, is a small cup-to-disc ratio, referred to as a “disc at risk”, characterized by crowding of the optic nerve head with a resultant compartment-like syndrome of the nerve [36].

The presentation of NAION is classically described as acute painless visual loss, often noted upon awakening, accompanied by an altitudinal visual field defect and dyschromatopsia. Frequently, it can progress over the ensuing days to weeks due to ongoing ischemia [37]. On examination, in addition to visual field deficits, patients may display a relative afferent pupillary defect and optic disc edema [36]. Moreover, erythrocyte sedimentation rate and C-reactive protein are normal, helping to rule out arteritic causes. During an acute episode of NAION, with optic disc edema, a disc-at-risk may not be identified on exam; however, it is likely to be present in the contralateral eye in around 80% [40].

Although variable, edema often resolves within six to 11 weeks; however, disc pallor is likely to persist [41]. Additionally, up to 40% of patients may experience some degree of recovery, but this is variable and less likely with more severe deficits [42]. Interestingly, there is a 20% risk for contralateral eye disease within five years; however, there is only around a 5% risk for recurrence in the ipsilateral eye. This is hypothesized to occur due to atrophy of the optic nerve head, relieving pressure [36,42]. At present, there are no available treatments for NAION, and treatment therefore relies on identifying and treating risk factors [36]. Only glucocorticoids have demonstrated some benefit with respect to visual outcomes, but the data are rather limited, and this is balanced with the risk for inadvertently exacerbating other risk factors such as glucose control [36].

The relationship between GLP-1 agonist therapy and NAION was first described in 2024 [8]. At present, it has been described with semaglutide, liraglutide, exenatide, and tirzepatide; however, not dulaglutide (Table 2). GLP-1 receptors are noted to be on the human neuronal eye cells [23]. From the limited literature available, it appears that the risk for developing NAION is greatest within the first year of therapy; the FDA Adverse Event Reporting system notes a median time to NAION development of 186 days [18]. It is rather peculiar as to why GLP-1 receptor agonists are associated with NAION; however, the drug class appears to target many of the risk factors that are associated with the disease. In addition, those who are taking GLP-1 receptor agonist therapy are already at heightened risk for developing NAION.

The exact pathophysiology remains unknown. An accepted theory, however, is that it is related to metabolic shifts rather than direct toxicity [36]. Rapid reduction of glucose with intensive diabetes therapy has previously been demonstrated to lead to a transient worsening of diabetic retinopathy with GLP-1 and insulin; additionally, the concept of ‘insulin neuritis’ is rather similar, linked to a rapid decline in hyperglycemia [43]. Unlike the diabetic retinopathy or insulin neuritis noted previously, however, NAION is not transient, with permanent vision loss. NAION is believed to occur in this setting from a rapid reduction in perfusion, such as with glucose reduction or weight loss (reducing plasma volume), coupled with GLP-1 influence upon the vascular tone leading to vasodilatation, ultimately resulting in hypoperfusion [36].

**Figure 2:**
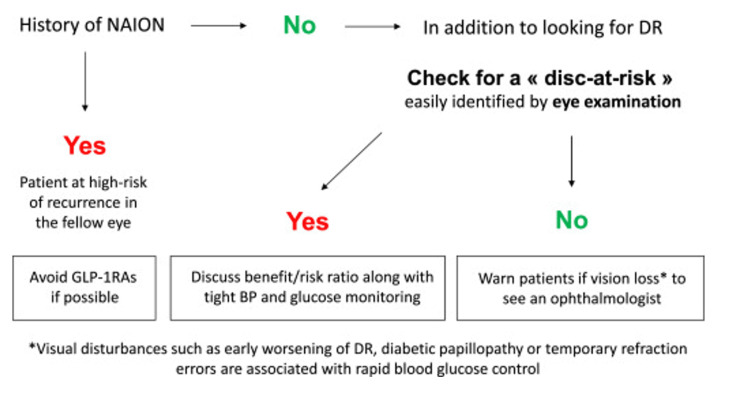
Algorithm for Prescribing Glucagon-Like Peptide-1 Receptor Agonist Therapy in Nonarteritic Anterior Ischemic Optic Neuropathy Permission obtained from Feldman-Billard [40] Abbreviations: BP = Blood Pressure; DR = Diabetic Retinopathy; NAION = Nonarteritic anterior ischemic optic neuropathy.

Due to the benefits greatly outweighing the small risk for NAION, the American Academy of Ophthalmology has issued a statement, for which they do not recommend discontinuing GLP-1 receptor agonist therapy, but recommend stopping the medication and seeking urgent medical attention should visual problems develop while on the medication [44]. Feldman-Billard recommends avoiding GLP-1 receptor agonist therapy in patients with a known history of NAION [40]. Additionally, Feldman-Billard recommends ocular examinations prior to commencing GLP-1 therapy to assess for a disc-at-risk; should GLP-1 be recommended despite the identification of a disc-at-risk, the authors advise a risk-benefit discussion with the patient, alongside gradual introduction of the medication with close attention paid to both the serum glucose and blood pressure [40]. Similar to the American Academy of Ophthalmology, Feldman-Billard advises urgent medical attention in the setting of any visual changes while on GLP-1 therapy (Figure 2) [40]. It is important to mention that most studies have excluded patients with pre-existing NAION; therefore, the risk for recurrence is unknown in patients with a pre-existing diagnosis who are exposed to GLP-1 therapy [40].

Our retrospective cohort study confirmed the association between GLP-1 receptor agonist therapy and the development of NAION over five years. Furthermore, our sensitivity analysis confirmed a moderately robust association. While statistically significant, however, the clinical significance is questionable. As demonstrated, the absolute risk increase is clinically minimal; translating this into clinical practice, the number needed to harm (NNH) is very high (NNH = 4,545). Our data demonstrated a risk of 0.087% for the development of NAION in patients with type 2 diabetes mellitus treated with GLP-1 therapy; in the clinical context, the risk for NAION is much lower than for cerebrovascular accidents, myocardial infarction, heart failure, chronic kidney disease, and all-cause mortality [45-52]. A further consideration is the reduced incidence of these comparative outcomes with GLP-1 therapy [45-52]. It is apparent that any potential risk is clinically small when weighed against the substantial, well-established benefits of GLP-1 therapy. Furthermore, withholding treatment on the basis of such a small risk would not be in the patient’s best interest.

Limitations

While we demonstrate a statistically significant (moderately robust) association between GLP-1 therapy and NAION, notable limitations exist. First and foremost, this was performed as a retrospective study, for which causality cannot be proven, but rather, an association is inferred. In addition, we utilized TriNetX Global Collaborative Network, which relies upon adequate ICD-10 coding, which can often be erroneous in medical records. As a notable example, there is no separate ICD-10 code for NAION (solely ION). In our analysis, we excluded patients with pre-existing NAION, and therefore, the risk for recurrence in patients with a prior history remains unknown. Although we included GLP-1 receptor agonists, a limitation was the inability to perform a sensitivity analysis stratifying the differing available GLP-1 therapeutics. Similarly, adherence, dosage, and duration of treatment were unable to be controlled for. Finally, while we included a five-year time window, we are unable to comment regarding the risk for NAION after more than five years of continued GLP-1 usage.

## Conclusions

In this retrospective cohort study, we demonstrate a statistically significant heightened risk for developing NAION after five years of treatment with a GLP-1 receptor agonist in patients with type 2 diabetes mellitus. Notably, however, within the literature, there are differing results amongst various studies, either proving or disproving a causal association. The suggestive but inconclusive association between GLP-1 RAs and NAION highlights critical gaps in knowledge and underscores the need for multidisciplinary research. Standardized NAION definitions and inclusion of diverse patient populations will improve generalizability. Overall, further research in a prospective fashion is required to investigate the underlying mechanism of NAION, assess the likelihood of recovery after GLP-1 discontinuation, and provide a comprehensive guideline for clinicians and patients to follow.
